# Occurrence and Antimicrobial Susceptibility Profiles of Multidrug-Resistant Aeromonads Isolated from Freshwater Ornamental Fish in Chiang Mai Province

**DOI:** 10.3390/pathogens9110973

**Published:** 2020-11-22

**Authors:** Banthita Saengsitthisak, Wasana Chaisri, Veerasak Punyapornwithaya, Raktham Mektrirat, Srikanjana Klayraung, John K. Bernard, Surachai Pikulkaew

**Affiliations:** 1 Graduate Program in Veterinary Science, Faculty of Veterinary Medicine, Chiang Mai University, Chiang Mai 50100, Thailand; banthitarx52@hotmail.co.th; 2 Faculty of Pharmacy, Payap University, Chiang Mai 50000, Thailand; 3 Department of Food Animal Clinic, Faculty of Veterinary Medicine, Chiang Mai University, Chiang Mai 50100, Thailand; wasana_kosa@hotmail.com (W.C.); pveerasak.r@gmail.com (V.P.); 4 Department of Veterinary Biosciences and Public Health, Faculty of Veterinary Medicine, Chiang Mai University, Chiang Mai 50100, Thailand; raktham.m@cmu.ac.th; 5 Epidemiology Research Center of Infectious Disease, Chiang Mai University, Chiang Mai 50100, Thailand; 6 Division of Biotechnology, Faculty of Science, Maejo University, Chiang Mai 50290, Thailand; srikanja@mju.ac.th; 7 Department of Animal and Dairy Science, The University of Georgia, Tifton, GA 31793-5766, USA; jbernard@uga.edu; 8 Research Center of Producing and Development of Products and Innovations for Animal Health and Production, Faculty of Veterinary Medicine, Chiang Mai University, Chiang Mai 50100, Thailand

**Keywords:** ornamental fish, multi-drug resistance, aeromonad, Chiang Mai

## Abstract

Antimicrobials are commonly used to prevent and treat disease in the ornamental fish industry. However, the indiscriminate and comprehensive overuse of unregulated antimicrobials without appropriate diagnostic examination could contribute to the development of antimicrobial-resistant strains of bacterial pathogens. Moreover, human infections caused by pathogens transmitted from fish or the aquatic environment are quite common. The frequent detection of antimicrobial resistance in ornamental fish and their environments are inevitable so as to decrease the transfer of antimicrobial-resistant bacteria from aquatic sources to other environments. This study evaluated the prevalence of common bacteria species and the antimicrobial susceptibility profile in ornamental fish that were sold in an ornamental fish shop in Chiang Mai, Thailand. *Aeromonas* spp. were the most dominant of the isolated species from the ornamental fish samples and accounted for 68.09% of the total. Other species detected included *Vibrio* spp., *Pseudomonas* spp., and *Citrobacter* spp. A high percentage of resistance to amoxicillin (93.75%), oxytetracycline (79.69%), and erythromycin (75.00%) was observed among the *Aeromonas* spp. The antimicrobial resistance information for ornamental fish is very limited, and the results from this study indicate that the *Aeromonas* spp. are highly resistant to several important antibiotics. The results suggest that additional steps should be taken to educate store owners to reduce the indiscriminate use of these antibiotics to decrease the antimicrobial resistance in ornamental fish to potentially improve public health.

## 1. Introduction

Ornamental fish-keeping is one of the most popular hobbies throughout the world today. Thus, the trade of ornamental fish globally has grown steadily [1,2]. More than 100 different countries supply ornamental fish, resulting in a large and diverse global industry valued at many millions of US dollars. Asia is the major supplier of ornamental fish, especially countries in South East Asia such as Malaysia and Thailand [2,3]. The common and economically important species of ornamental freshwater fish are goldfish (*Carassius auratus*), koi carp (*Cyprinus carpiokoi*), red swordtail (*Xiphophorus hellerii*), and Zebrafish (*Danio rerio*) [1,4,5,6]. The ornamental aquaculture faces problems, especially from bacterial infections [4,7]. A bacterial disease outbreak results in increased mortality in the aquaculture and ornamental fish populations, causing an economic loss to producers [8,9]. Environmental factors that negatively influence the immune function and lead to infections in fish such as water quality, temperature, and pH, along with an unhygienic care and poor feed quality, also contribute to an increased disease occurrence [6,10].

Generally, antimicrobials are administered by the veterinarian and owner or retailer to control and eradicate bacterial infections in ornamental fish [2,6]. During the transport of ornamental fish, antimicrobials are normally added to the water to suppress the growth of potential pathogens. This management practice has been used widely for more than thirty years [2,11,12]. However, indiscriminate use, comprehensive overuse, and the lack of proper clinical examination before using antimicrobials or treating infected ornamental fish with antimicrobials increases the potential for antimicrobial resistance of both commensal and pathogenic organisms [1,3,6,13]. This has reduced the effectiveness of treatments normally used for bacterial infections, making it more difficult and expensive to treat a disease, and causes additional environmental hazards, including the contamination of soil and water, resulting in the development of additional resistant bacteria strains [1,6,14]. These sources could be a reservoir of antimicrobial resistance genes. It includes resistance genes in antimicrobial producers, and precursor genes evolve to act as resistance elements. This gene pool has been recently named the “resistome”.

The antimicrobial resistance in pathogenic bacteria is a serious problem in the aquaculture industry and continues to be a significant issue for humans and other animals that become infected with these pathogens [1,2,12]. Infections caused by antimicrobial-resistant bacteria and the spreading genes of these antibiotic-resistant pathogens are not only an industry issue because of the potential economic losses but, also, represent a major public health issue [15,16,17]. Limited information on the prevalence of the antimicrobial resistance of bacteria isolated from the ornamental fish industry is available. Moreover, many of the common bacterial isolates are potentially zoonotic [3,6]. Interestingly, researchers investigating bacterial infections in humans also isolated the same pathogens from both the water and fish of the infected patient’s aquarium [3]. Thus, additional research is needed to evaluate the potential antibiotic resistance and potential transfer of resistance in common bacterial strains that cause infections in tropical and ornamental fish [2]. The primary objective of this study was to measure the prevalence of pathogenic bacterial species, and the secondary objective was to describe the potential antimicrobial resistance pattern of common bacterial species isolated from ornamental fish sold in fish shops located in Chiang Mai, Thailand.

## 2. Results

### 2.1. Occurrence of Pathogenic Bacteria from Ornamental Fish

In this study, 94 bacterial isolates from 80.21% (77/96) of the sampled freshwater ornamental fish were identified based on their morphological properties, Gram staining, and a series of biochemical tests. The occurrence of pathogenic bacterial isolates identified in freshwater ornamental fish is shown in Table 1. The most abundant pathogenic bacteria were *Aeromonas* spp., accounting for 68.09% of the total bacterial species, followed by *Vibrio* spp. (12.77%), *Viridans streptococci* (5.32%), and the others (10.64%). In addition, three probable isolates of aeromonads, including *A. sobria* (43.62%), *A. hydrophila* (19.15%), and *A. caviae* (5.32%), were identified.

### 2.2. Antimicrobial Susceptibility Profiles of Bacteria Isolates

Antimicrobial susceptibility was determined for the 64 strains of aeromonads. The isolated bacteria exhibited variable resistance rates to 12 antimicrobial agents (Table 2). Interestingly, the highest resistance was observed for amoxicillin (93.75%), followed by oxytetracycline (79.69%), erythromycin (75.00%), sulfamethoxazole/trimethoprim (46.88%), ciprofloxacin (40.63%), enrofloxacin (25.00%), norfloxacin (25.00%), gentamicin (17.19%), amikacin (12.50%), nitrofurantoin (7.81%), and chloramphenicol (6.25%), respectively. None of the isolates were resistant to ceftazidime.

**Figure 1:**
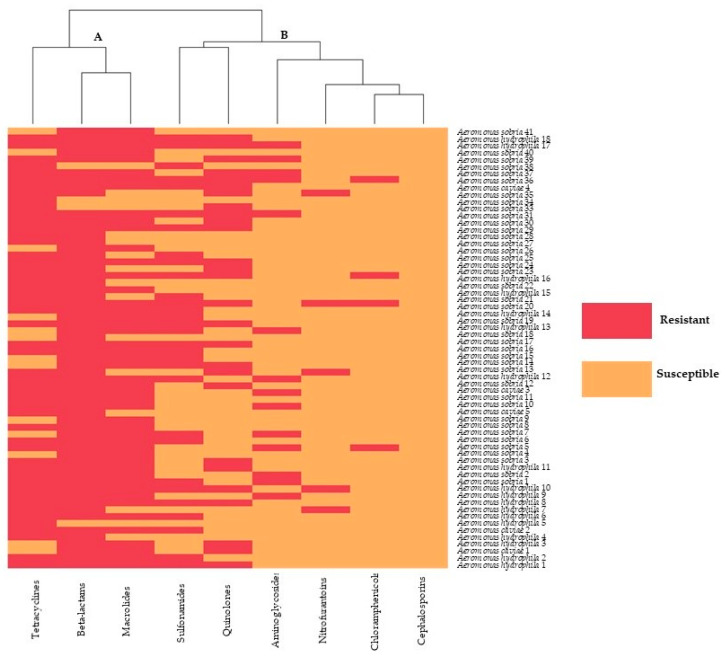
Heat map showing the antimicrobial susceptibility profile of aeromonad isolates from ornamental fish. Columns represent individual antibiotics, and rows represent aeromonad strains. Red blocks indicate resistance, and orange blocks indicate susceptibility to the antibiotics.

The heat map (Figure 1) illustrates the hierarchical clustering of Aeromonas isolates according to their phenotypical profile (antimicrobial resistance). There were two predominant clusters, including cluster A and cluster B. Red blocks represent the resistance, and orange blocks indicate susceptibility to the nine groups of antimicrobial agents, which included tetracyclines, beta-lactams, macrolides, sulfonamides, quinolones, aminoglycosides, nitrofurantoins, chloramphenicols, and cephalosporins. Accordingly, the antimicrobial susceptibility of the aeromonad strains was divided into two groups. Group A were resistant to tetracyclines, beta-lactams, and macrolides. Group B were susceptible to sulfonamides, quinolones, aminoglycosides, nitrofurantoins, chloramphenicols, and cephalosporins.

### 2.3. Determination of MIC and MBC

The MIC (minimal inhibitory concentration) and MBC (minimal bactericidal concentration) values obtained were important parameters for reporting the results of susceptibility testing for the multiple isolates of the aeromonad species. The results indicate that the aeromonad isolates were inhibited by 11 antimicrobial agents with different MIC_50_ and MIC_90_ values (Table 3). The MIC_50_ of amoxicillin, oxytetracycline, and erythromycin exhibited the highest resistance at 1073.40, 34.97, and 52.91 µg/mL, respectively. Moreover, the aeromonads tested were inhibited at MIC_90_ concentrations of 2222.26 µg/mL for amoxicillin, 149.26 µg/mL for oxytetracycline, and 92.13 µg/mL for erythromycin. As reported in Table 4, the MBC_50_ and MBC_90_ values indicated bactericidal concentrations for 11 of the antimicrobial agents against aeromonads. The MBC_50_ of amoxicillin, sulfamethoxazole, and ciprofloxacin were highest at 1873.50, 713.43, and 154.60 µg/mL, respectively. The MBC_90_ of amikacin (38.77 µg/mL) exhibited potential bactericidal activity against aeromonads, while the MBC_90_ of amoxicillin reached 3873.50 µg/mL.

### 2.4. Antimicrobial Capacity

The MIC and MBC of 11 antimicrobial agents varied, as described above. The ratio of MBC/MIC was used to interpret the antimicrobial activity of the drugs. The bactericidal effect of all 11 antimicrobial agents for *Aeromonas* spp. was defined as MBC_90_/MIC_90_ (Table 5). In addition, the MBC_50_/MIC_50_ ratio of three antimicrobials: oxytetracycline, ciprofloxacin, and chloramphenicol was used to characterize the bacteriostatic activity against aeromonads. Furthermore, the prevalence of amoxicillin tolerance was highest among the isolates tested at 67.19% (43/64).

## 3. Discussion

In the present study, ninety-four isolates of nine different genera were obtained from the 96-ornamental fish sampled. *Aeromonas* spp. (68.09%) was the predominant isolate identified in the sample population, in agreement with the results of previous trials that identified infectious bacterial genera in ornamental fish [3,5,18,19,20,21,22]. Aeromonads are common in normal flora of water resources but could also develop as an opportunistic bacterial pathogen of aquatic animals when their immune systems are weakened [19,23], increasing the mortality of freshwater fish species [23,24]. Aeromonads have been correlated with a wide range of infections observed in freshwater animals, with the most frequently isolated species being *A. hydrophila*, *A. sobria*, *A. caviae,* and *A. veroni*. Moreover, these bacteria could also be pathogenic for human beings and have been incriminated as a possible cause of organ infections in humans, such as the gastrointestinal tract, skin, urinary tract, and muscle infections [4,5,24,25]. In addition, three species of Aeromonas, including *A. hydrophila*, A*. sobria,* and *A. caviae*, are the most common bacterial causal agents typically correlated with septicemia in fish that also pose a potential risk for public health [21,23,26]. Interestingly, a 10-year retrospective survey of emerging pathogens from patients at a tertiary-care hospital in Hungary found resistance trends with a high prevalence of *Aeromonas* spp. (97.9%) and *Plesiomonas shigelloides* (2.1%), respectively [27]. Moreover, *Aeromonas* spp. (68.09%), *Vibrio* spp. (12.77%), *Viridans streptococci*. (5.32%), *Pseudomonas* spp. (2.53%), and *Citrobacter* spp. (1.27%) were also observed in the random sample of ornamental fish obtained in our current study. These bacteria were also isolated from ornamental fish in previous studies [3,5,24,26,28].

Previous studies on the prevalence of antimicrobial resistance in ornamental fish have also reported a high resistance to many antimicrobials, including oxytetracycline, nitrofurans (e.g., furazolidone), sulfonamides, and oxalinic acid, which is most likely a result of their common use in the domestic fish trade [2,12]. In our study, most of the *Aeromonas* isolates were highly resistance to amoxicillin (93.75%), oxytetracycline (79.69%), and erythromycin (75.00%).

Our results are in agreement with many previous studies that reported resistance to amoxicillin, which is classified as a beta-lactam antimicrobial agent. Resistant *Aeromonas* spp. have been identified in various aquatic sources, including sea trout in Latvia; ornamental fish in India; goldfish in Korea and Thailand (100% amoxicillin resistance); diseased ornamental fish in Thailand (92.31% were resistant to penicillin and amoxicillin, and 83.33% were resistant to ampicillin); skin and water from imported ornamental fish in Portugal (95% were resistant to amoxicillin, 96% resistant to carbenicillin, and 94% resistant to ampicillin); fish and prawn in south India (100% were resistant to methicillin); and water and fish from freshwater fish farms in Syria (more than 75% were resistant to amoxicillin) [6,13,23,24,26,29,30,31]. Beta-lactam tolerance in bacteria is frequently due to the presence of the beta-lactamase enzyme, which is produced by aeromonads and many strains of Gram-negative bacilli. Furthermore, resistance to these antimicrobial agents is chromosomally mediated, although it is frequently caused by plasmids or integrons. The increased level of antimicrobial drug resistance is also driven by the frequent use of antimicrobials to treat diseases in ornamental fish [22]. In many organisms, especially aeromonads, beta-lactamase genes are intrinsic, and their expression has led to a beta-lactam challenge [32,33].

High resistance rates were also observed against oxytetracycline in *Aeromonas* spp. Oxytetracycline was the second of the tetracycline group of antimicrobials to be discovered. It is typically used as an antimicrobial, either prophylactically or for treatment against bacterial infections in aquaculture farms because of its broad-spectrum antimicrobial properties. Thus, bacteria seem to be increasingly resistant to oxytetracycline [34]. Hossain et al. [6] reported that every *Aeromonas* strain isolated from healthy zebrafish in Korea was resistant to oxytetracycline and tetracycline (100% and 74.42%, respectively). These results are in agreement with the study of *Aeromonas* spp. isolated from sea trout in Latvia in which more than 90% of bacteria were resistant to oxytetracycline [13]. Similar results were reported by Jongjareanjai et al. [30], Kanchan et al. [23], and Dood N. [26], whose data indicated that 84.62%, 50.00%, and 65% of *Aeromonas* spp. were resistant to oxytetracycline, and 81.82%, 50.00%, and 53.00% were resistant to tetracycline, respectively. Other studies examining antimicrobial resistance in the aquaculture found occurrences of oxytetracycline-resistant *Aeromonas* spp. similar to our results. Samples of skin and water from imported ornamental fish in Portugal revealed 80% tetracycline resistance for *Aeromonas* spp. [31]. This was not surprising, as the isolated bacteria from fish were, in most cases, resistant to antimicrobials commonly used in the ornamental fish trade to treat the infections of ornamental fish, such as amoxicillin and oxytetracycline. Interestingly, these researchers also observed a resistance to gentamicin and amikacin of 17.19% and 12.50%, respectively [31]. In contrast to our results, Hossain et al. [6], Revina et al. [13], John et al. [24], Stratev et al. [22], Dood N. [26], Vivekanandhan et al. [29], and Odeyemi et al. [35] reported that the least resistance was to aminoglycosides.

On the other hand, 93.75% of the Aeromonad strains exhibited high levels of resistance against chloramphenicol. This is in agreement with the results of John et al. [24], Jongjareanjai et al. [30], Dias et al. [31], and Vivekanandhan et al. [29], who observed 100%, 87%, 59.09%, and 95.6% resistance, respectively. In contrast, *Aeromonas* spp. isolated from goldfish in Thailand and aquatic sources including seawater, bivalves, and sea cucumbers in Malaysia were 33.33% and 20.8% resistant to chloramphenicol [23,35]. Moreover, a complete susceptibility to ceftazidime was observed in all aeromonad isolates tested in our current study, which was in contrast to the results reported by John et al. [24] in their research in India. In their study, aeromonads isolated from ornamental fish and water samples were reported to have 18.96% and 8.00% resistance to ceftazidime [24].

The MIC and MBC were defined as the lowest concentrations of antimicrobials that prevented the visible growth and killed aeromonads. The results in this study were presented as MIC_50_, MIC_90_, MBC_50_, and MBC_90_ values based on the calculations described in the Materials and Methods. The point of interception, the median MIC_50_ and MBC_50_, and the 90th percentile MIC_90_ and MBC_90_ were identified as the values for the application of treatment decision-making for diseased fish. The antimicrobial with the highest MIC_50_, MIC_90_, MBC_50_, and MBC_90_ values was amoxicillin. Amoxicillin resistance was highest for aeromonads, as discussed above. The MIC_50_ and MIC_90_ of amoxicillin were 1073.40 and 2222.26 µg/mL, respectively. Moreover, the MBC_50_ and MBC_90_ of amoxicillin were 1873.50 and 3873.50 µg/mL, respectively. These results were higher compared to Lamy et al. [36], who reported MIC_50_ and MIC_90_ of amoxicillin in aeromonad isolates of more than 128 µg/mL. The MIC_90_ of *A. caviae*, *A. vernii*, and *A. hydrophilla* parent isolates to ampicillin were 64, 64, and 128 µg/mL, respectively [33]. The MIC_90_ to ampicillin of *A. caviae* was 1024, and the MIC_90_ of *A. vernii* and *A. hydrophilla* was more than 1024 µg/mL [33]. Aeromonads exhibited the greatest resistant to amoxicillin, as confirmed by the highest percentage of tolerance (67.19%). However, the ratio of MBC_90_/MIC_90_ for amoxicillin (ratio = 1.74) indicates a bactericidal activity that should kill this strain. Gentamycin and amikacin are classified as aminoglycoside antimicrobial agents and have been reported as least tolerant in aeromonad strains [22]. The MIC_50_, MIC_90_, MBC_50_, and MBC_90_ of gentamicin and amikacin were 18.16, 11.29, 43.31, and 34.41 µg/mL, respectively. These data are in contrast to the results observed for aeromonad strains in the USA and France [3,36]. The MIC_50_ and MIC_90_ values to gentamicin and amikacin in France were 0.5, 2, 1, and 4 µg/mL, respectively [36]. The *A. hydrophila* recovered from freshwater ornamental fish species imported into Oregon, USA had MIC values of 4 µg/mL [3]. On the other hand, the ratios of MBC_50_/MIC_50_ and MBC_90_/MIC_90_ of gentamicin and amikacin implied that these antimicrobials exhibited bactericidal activity in those studied.

As mentioned above, some bacteria isolated from food fish and ornamental fish are known to also be pathogenic to humans. In this situation, health should be considered as the broader concept, including animal, human, and environmental health, as outlined in the One Health approach adopted by many international health institutions [37]. Our data and previous reports indicate that individuals with immunosuppression should be aware of the potential for the zoonotic transfer of pathogens that have antimicrobial resistance when interacting with the water, the fish, or the contaminated environment. Common pathogens identified in freshwater ornamental fish were *Aeromonas* spp., whereas *Vibrio* spp., *Pseudomonas* spp., and *Citrobacter* spp. were not frequently observed colonizing fish or water in our study. Our results confirm that ornamental fish carry a diverse bacterial population, including pathogens common to both fish and humans. Moreover, many bacterial strains have been isolated without any apparent disease signs displayed in the fish. Pathogens and antimicrobial resistance could be transferred from one host to another. Due to this, it was important to acquire information about the antimicrobial resistance profiles of bacteria isolated from ornamental fish.

The antimicrobial resistance observed in our results is most likely associated with the overuse of antimicrobials to prevent and or treat diseases in ornamental fish and suggest that additional education is needed in the ornamental fish industry to reduce the incidence of antimicrobial resistance. The use of an ineffective antimicrobial as a therapy may delay the recovery of diseased fish and could result in increased mortality. Information on the susceptibility to antimicrobials of the colonizing bacteria can help improve the best practices among veterinary and medical practitioners. However, the use of antimicrobials should be more targeted and prudent. This study shows that veterinarians and the ornamental fish industry should recognize the high degree of antimicrobial resistance seen in bacteria colonizing these fish.

## 4. Materials and Methods

### 4.1. Sample Collection

**Figure 2:**
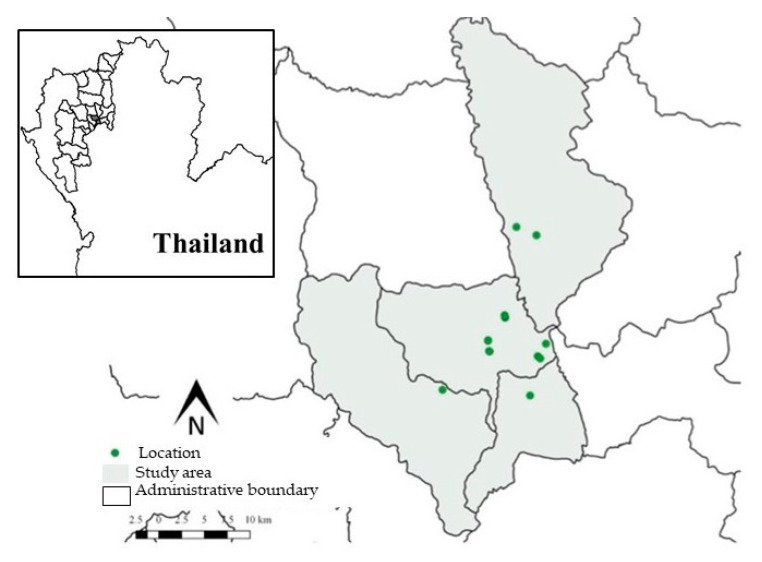
Location of the distribution of 19 ornamental fish shops (green dots) in the Chiang Mai Province sampled for the study (figures obtained and modified from a free media repository).

A total of 96 freshwater ornamental fish were collected and 94 tanks sampled in six ornamental fish shops located in Chiang Mai Province in Northern Thailand from July 2016 to June 2017. These shops represent a subset of registered ornamental fish shops in Chiang Mai identified in a 2016 survey (https://www.fisheries.go.th/fpo-chiangmai/web2/, accessed on 10 June 2016). The survey indicated a total of 150 tanks that were located in these ornamental fish shops based on interviews with the shop owners. Figure 2 presents the locations within Chiang Mai of the 19 registered ornamental fish shops sampled in this study created using Quantum Information System (QGIS) version 2.18.28. The sample size for determining the prevalence was calculated using R (version 3.62) [38] and the epiR package [39]. The prevalence was set at 50%, and the accepted absolute error or precision was set at 0.1%, with the accepted absolute error or precision set as 0.1%. Selection of the individual ornamental fish shops to collect fish was based on accessibility and convenience. Moreover, the shops selected for the study represented 80% of all the fish shops in Chiang Mai. The number of tanks within each shop sampled ranged from 2 to 20. We randomly selected 3 tanks from each shop, and 1 fish from each tank was randomly collected. The number of each species collected for the study and their body weight and total body length (average ± SD) included: 72 goldfish (*Carassius auratus*) (7.61 ± 0.75 g and 7.33 ± 0.36 cm), 18 koi (*Cyprinus carpio* koi) (24.76 ± 2.76 g and 14.50 ± 1.76 cm), and 6 red swordtails (*Xiphophorus hellerii*) (0.88 ± 0.18 g and 6.54 ± 0.48 cm^2^).

Random sampling of fish presenting at least one clinical sign of bacterial infection (skin ulcerations, abdominal hemorrhages on skin or fins, and fin rot) were collected. The sampled fish were rinsed 2 to 3 times with water and individually placed into sterilized polyethylene bags for transport to the laboratory for testing on the same day. This study was ethically approved by the animal ethics committee of the Faculty of Veterinary Chiang Mai University (No. R1/2559).

### 4.2. Bacterial Isolation, Identification, and Biochemical Characterization

All equipment was sterilized with 100% ethanol or autoclaved prior to the collection of liver and spleen samples from each of the fish. The technician wore sterilized gloves and used 70% ethanol to sanitize all surfaces and equipment prior to sample collection to prevent any potential contamination of samples. The samples were inoculated in 5% sheep blood agar plates and MacConkey agar plates, which are a general-purpose medium used for aquatic animal specimens to improve the isolation of some organisms [40]. The plates were incubated at 27 °C for 24–48 h for further analysis [40].

Bacterial isolates were morphologically identified via colony morphology, Gram staining, and motility test. Further identification and classification of the isolates into species were preformed using oxidase, starch hydrolysis, indole, and H_2_S tests [9,40]. A total of 94 bacterial isolates were selected from the pure cultures and maintained on tryptic soy agar (TSA) plates. The 20% glycerol stock cultures were stored at −80 °C for long-term preservation.

### 4.3. Antimicrobial Susceptibility Test

Antimicrobial susceptibility was determined by the disc diffusion method in Mueller-Hinton agar plates according to the National Committee for Clinical Laboratory Standards 1997 guideline [41]. Isolates were streaked on 5% sheep blood agar plates and incubated at 27 °C for 24–48 h. Then, 4 to 5 colonies were inoculated in a 5-mL tube of tryptic soy broth (TSB) and incubated for 4–6 h. at 27 °C to obtain a bacterial suspension that corresponded in turbidity to a 0.5-McFarland standard. Using a sterile swab, the entire surface of a Mueller-Hinton agar plate was inoculated with the bacteria suspension. Antimicrobial classes used to detect the antimicrobial susceptibility of bacterial isolates included amikacin 30 µg (AK), amoxicillin 10 µg (AML), ceftazidime 30 µg (CAZ), chloramphenicol 30 µg (C), ciprofloxacin 5 µg (CIP), enrofloxacin 5 µg (ENR), erythromycin 15 µg (E), gentamicin 10 µg (GN), nitrofurantoin 300 µg (N), norfloxacin 10 µg (NOR), oxytetracycline 30 µg, (OT), and sulfamethoxazole-trimethoprim 25 µg (SXT). All discs were obtained from Oxoid^®^ (Oxoid Limited, Hampshire, England). After 24-h incubation at 30 °C, bacterial isolates were classified as susceptible (S), intermediately resistant (I), or resistant (R) based on the size of the zone of the bacteria growth inhibition, as described by the Clinical and Laboratory Standards Institute 2010 [42]. Bacterial isolates were classified into groups: sensitivity group and resistant group (intermediate was interpreted as resistant) for a heat map analysis by R (version 3.42) to illustrate a hierarchical clustering of bacterial isolates based on the susceptibility pattern.

### 4.4. Minimum Inhibitory Concentration (MIC) and Minimal Bactericidal Concentration (MBC)

The MIC and MBC of 12 antimicrobials against bacteria isolates were performed by a broth micro-dilution assay technique in a 96-well microtiter plate in accordance with the guidelines of the Clinical and Laboratory Standards Institute, document M45, and drop plate techniques, respectively [30,31,43]. The isolates that classified as intermediately resistant (I) or resistant (R) by the disc diffusion method were evaluated for MIC and MBC values. Mueller-Hinton broth containing the antimicrobial (200 µL) was added to the first column well, and Mueller-Hinton broth (100 µl) was pipetted to the others of the 96-well microtiter plates. A series of two-fold dilution of the antimicrobial was dispersed in the columns of the wells. Finally, ten microliters (10 µL) of bacterial suspension equivalent to 0.5 McFarland units in sterile saline was added to each well and incubated at 27 °C for 18–24 h. MIC was determined as the least concentration of the antimicrobial that inhibited the growth of the test bacteria. All assays were carried out in three replicates. The MBC of the antimicrobials was determined using the Kowser et al. (2009) method [44], with the modification that 5 µL of the medium was taken from the wells that displayed negative visible growth in the MIC assay and sub-cultured on a Mueller-Hinton plate and incubated at 27 °C for overnight. The MBC was determined as the lowest concentration of the antimicrobial that did not allow any bacterial growth on the surface of the agar plates. All assays were carried out in triplicate**.** MIC and MBC distributions were described using the median MIC_50_ and MBC_50,_ and the 90th percentile MIC_90_ and MBC_90_ [36,45]. These values were determined by plotting the cumulative percentage on the Y-axis against the concentration (µg/mL) on the X-axis for all MIC and MBC for each individual antimicrobial evaluated. The trend lines of the experimental cumulative curves were calculated using the 6th degree polynomial approximation of the experimental data. The regression equations and the correlation coefficients were also calculated using R software (version 3.42) [46].

The antimicrobial activity of the bacterial strains can be described by MIC and MBC. Antimicrobial activity was determined by the ratio of MBC/MIC. If the ratio MBC/MIC was ≤ 2, the effect was evaluated as bactericidal, whereas if a ratio MBC/MIC ≥ 4, the effect was evaluated as bacteriostatic [47,48]. Tolerance, defined as an MBC/MIC ratio of 32 or an MBC/MIC ratio of 16 with an MBC in the resistant range, was evaluated, and the overall tolerance rates for each antimicrobial were calculated [49].

### 4.5. Statistical Analysis and Data Reconfiguration

MIC and MBC distributions were described using the median MIC_50_ and MBC_50_, and the 90th percentile MIC_90_ and MBC_90_ were summarized using R (version 3.62) [38] and epiR package [39] to calculate as the median (IQR: interquartile range).

## 5. Conclusions

This research indicates aeromonads as the predominant bacteria isolated in ornamental fish sold in stores for hobbies. The aeromonads also exhibited a high level of resistance to amoxicillin, oxytetracycline, and erythromycin. The antimicrobial resistance is not only a problem for the ornamental fish industry; it is also a potential global public health issue and an ecological problem, as defined by the complex interactions involving diverse microbial populations affecting the health of humans, animals, and the environment. It makes sense to address the resistance problem by taking this complex and ecological nature into account using a coordinated, multi-sectoral approach, such as One Health. However, the antimicrobial resistance data from ornamental fish industries in Thailand and elsewhere is infrequently assessed. The information from our current research can be useful for increasing educational efforts related to the prevention of resistance and decreasing the severity of the antimicrobial resistance problem. For a deeper understanding into the mechanisms that bacteria exhibit to protect themselves from antimicrobials and bacterial genes involved with resistance, further studies should concentrate on the molecular characterization of the bacterial isolates identified to have resistance.

## Figures and Tables

**Table 1:** The identification of bacterial isolates from the liver and spleen of ornamental fish in Chiang Mai Province and their frequency of occurrence during July 2016 to June 2017.

Bacteria	Bacterial Isolates, *n* (%)
*Aeromonas sorbria*	41 (*43.62*)
*Aeromonas hydrophila*	18 (*19.15*)
*Aeromonas caviae*	5 (*5.32*)
*Vibrio cholerae*	8 (*8.51*)
*Vibrio minicus*	4 (*4.26*)
*Pseudomonas aeruginosa*	2 (*2.13*)
*Citrobacter freundii*	1 (*1.06*)
*Viridans streptococci*	5 (*5.32*)
*Plesiomonas shigelloides*	3 (*3.19*)
*Salmonella enterica*	3 (*3.19*)
*Edwardsiella tarda*	2 (*2.13*)
*Enterobacter* spp.	2 (*2.13*)

**Table 2:** Summary of the antibiotic resistance profiles of pathogenic aeromonads isolated from ornamental fish.

Antimicrobial Agents	Concentration (µg)	Bacterial Isolates			
		*Aeromonas sorbria* (*n* = 41)	*A. hydrophila* (*n* = 18)	*A. caviae* (*n* = 5)	Total (*n* = 64)
amoxicillin (AML)	10	38	17	5	60 (93.75%)
oxytetracycline (OT)	30	32	15	4	51 (79.69%)
erythromycin (E)	15	31	13	4	48 (75.00%)
sulfamethoxazole- trimethoprim (SXT)	25	17	11	2	30 (46.88%)
ciprofloxacin (CIP)	5	17	7	2	26 (40.63%)
enrofloxacin (ENR)	5	10	5	1	16 (25.00%)
norfloxacin (NOR)	10	12	4	0	16 (25.00%)
gentamicin (GN)	10	7	3	1	11 (17.19%)
amikacin (AK)	30	6	1	1	8 (12.50%)
nitrofurantoin (N)	300	3	2	0	5 (7.81%)
chloramphenicol (C)	30	0	4	0	4 (6.25%)
ceftazidime (CAZ)	30	0	0	0	0 (0%)

**Table 3:** Minimum inhibitory concentrations required to inhibit the growth of 50% and 90% of the pathogenic aeromonads isolated from ornamental fish.

Antimicrobial Agents	Minimal Inhibitory Concentration of Bacterial Isolates (µg/mL)		
	MIC_50_	MIC_90_	Median (IQR) ^1^
amoxicillin (AML)	1079.40	2222.26	2048 (512–4096)
oxytetracycline (OT)	34.97	149.26	512 (64–2048)
erythromycin (E)	52.91	92.13	512 (128–1024)
sulfamethoxazole (S)	321.25	571.25	2048 (512–4096)
ciprofloxacin (CIP)	57.53	268.05	256 (64–2048)
enrofloxacin (ENR)	27.03	71.48	128 (16–512)
norfloxacin (NOR)	56.20	101.15	256 (128–512)
amikacin (AK)	11.29	34.41	96 (28–320)
gentamicin (GN)	18.16	43.31	128 (32–512)
nitrofurantoin (N)	4.28	63.99	128 (32–512)
chloramphenicol (C)	4.43	45.24	64 (16–256)

^1^ Median (interquartile).

**Table 4:** Minimum bactericidal concentrations required to kill 50% and 90% of the pathogenic aeromonads isolated from ornamental fish.

Antimicrobial Agents	Minimal Bactericidal Concentration Bacterial Isolates (µg/mL)		
	MBC_50_	MBC_90_	Median (IQR) ^1^
amoxicillin (AML)	1873.50	3873.50	2048 (512–4096)
oxytetracycline (OT)	120.29	224.73	1024 (256–2048)
erythromycin (E)	85.64	148.14	512 (256–1024)
sulfamethoxazole (S)	713.43	1284.86	2048 (1024–8192)
ciprofloxacin (CIP)	154.60	354.60	512 (128–3072)
enrofloxacin (ENR)	51.28	110.10	256 (32–1024)
norfloxacin (NOR)	103.73	192.62	512 (128–1024)
amikacin (AK)	18.97	38.77	128 (32–512)
gentamicin (GN)	30.05	59.25	192 (64–512)
nitrofurantoin (N)	4.28	63.99	128 (32–512)
chloramphenicol (C)	34.80	66.55	256 (128–512)

^1^ Median (interquartile).

**Table 5:** MBC_50_/MIC_50_ and MBC_90_/MIC_90_ ratios and percentages of tolerance of *Aeromonas* spp. isolates to 11 antimicrobial agents.

Antimicrobial Agents	Bacterial Isolates				
	MBC_50_/MIC_50_ (µg/mL)		MBC_90_/MIC_90_ (µg/mL)		% Tolerance ^2^
	Ratio	Value ^1^	Ratio	Value	
Amoxicillin (AML)	1873.50/1079.40	1.74	3873.50/2222.26	1.74	67.19
oxytetracycline (OT)	120.29/34.97	3.44	224.73/149.26	1.51	57.81
erythromycin (E)	85.64/52.91	1.62	148.14/92.13	1.61	53.13
sulfamethoxazole-(S)	713.43/321.25	2.22	1284.86/571.25	2.25	35.94
ciprofloxacin (CIP)	154.60/57.53	2.69	354.60/268.05	1.32	29.69
enrofloxacin (ENR)	51.28/27.03	1.90	110.10/71.48	1.54	17.19
gentamicin (GN)	30.05/18.16	1.74	59.25/43.31	1.37	17.19
norfloxacin (NOR)	103.73/56.20	1.85	192.62/101.15	1.90	15.63
amikacin (AK)	18.79/11.29	1.68	38.77/34.41	1.13	12.50
nitrofurantoin (N)	4.28/4.28	1.00	63.99/63.99	1.00	6.25
chloramphenicol (C)	34.80/4.43	7.86	66.55/45.24	1.47	6.25

^1^ Values ≤ 2 were considered to have bactericidal activity, and values ≥ 4 were considered to have bacteriostatic activity. ^2^ Percent tolerance was defined as an MBC/MIC ratio of ≥32 or an MBC/MIC ratio of ≥16 when the MBC was greater than or equal to the breakpoint for resistance.
